# A simple judgment method for joint action of antibacterial agents on bacterial resistance

**DOI:** 10.1016/j.mex.2022.101700

**Published:** 2022-04-19

**Authors:** Liang Tang, Danqing Tong, Yulian Zhang, Jiajun Wang, Haoyu Sun

**Affiliations:** Key Laboratory of Organic Compound Pollution Control Engineering (MOE), School of Environmental and Chemical Engineering, Shanghai University, 333 Nanchen Road, Shanghai 200444, China

**Keywords:** Bacterial resistance, Antibacterial agents, Joint action, Mutation unit, Conjugative transfer unit

## Abstract

The severe pollution of bacterial resistance induced by the wide and even indiscriminate use of antibacterial agents has posed serious threats to human health and ecological safety. Furthermore, the combined effects of antibacterial agents have a closer relationship with the pollution of bacterial resistance than single antibacterial agent. However, there is little information regarding how multiple antibacterial agents interplay to induce bacterial resistance. Here, we developed a simple judgment method with five basic procedures for the joint action of antibacterial agents on bacterial resistance, involving toxicity determination, mutation frequency determination, conjugative transfer frequency determination, dose-response relationship fitting, key parameters obtaining, and joint resistance action judgment. This proposed approach was validated through investigating the joint resistance action between silver nanoparticle (AgNP) and 1-pyrrolidino-1-cyclohexene (1P1C, a kind of quorum sensing inhibitors). According to the procedures, the mutation unit and conjugative transfer unit for the AgNP-1P1C mixture were calculated to be 64.27 and 5.10, respectively, indicating the antagonism for their joint resistance action. This method can not only benefit the mechanistic explanation for how mixed antibacterial agents stimulate the bacterial resistance, but also guide the environmental risk assessment and clinical use of combined antibacterial agents in the related fields.

• We present a novel method to judge the joint resistance action of antibacterial agents, taking the emergence and dissemination of antibiotic resistance genes into account.

• Toxicity determination can help to design the mixtures of antibacterial agents and confirm the appropriate test concentration range of antibacterial agents used in mutation frequency and conjugative transfer frequency determination.

• The mutation unit and conjugative transfer unit were proposed according to the toxic unit in the judgment of joint toxic action.

## Graphical abstract

**Figure:**
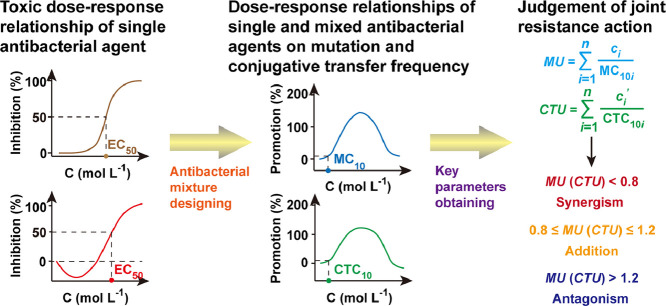

## Specifications table

**Table:** 

Subject Area:	Environmental Science
More specific subject area:	Environmental Toxicology
Method name:	Joint resistance action of antibacterial agents
Name and reference of original method:	Hormetic dose-responses for silver antibacterial compounds, quorum sensing inhibitors, and their binary mixtures on bacterial resistance of *Escherichia coli*, Shen et al. 2021 (doi: 10.1016/j.scitotenv.2021.147464)
Resource availability:	Non

## Method details

### Background

Bacterial resistance, as a global health crisis, has aroused wide public concern over the last few decades [1]. The wide and even indiscriminate use of antibiotics and some other antibacterial compounds in medicine, animal husbandry, and family practice are considered to cause the severe pollution of bacterial resistance [2,3]. Bacteria that possess resistance to antibacterial agents make the prevention and control of infectious diseases difficult, which pose serious threats to human health and ecological safety [4]. Hence, it is necessary to investigate how antibacterial agents result in bacteria developing resistance.

One of the most important conditions for bacteria developing resistance is to obtain antibiotic resistance genes (ARGs). As emerging contaminants with environmental persistence, ARGs have been found in various environmental media, featured by high abundance and diversity, which are regarded to closely relate with the pollution of bacterial resistance [5]. Mutation and horizontal gene transfer are the main ways for bacteria acquiring ARGs, which are usually used to characterize the emergence and dissemination of ARGs [6,7]. Previous studies have observed the facilitation of many single antibacterial agents on the mutation and horizontal gene transfer, which indicated that these agents could induce bacterial resistance through triggering hormetic effects on bacteria acquiring ARGs [8,9]. For example, sulfonamides could significantly promote the mutation frequency and RP4 plasmid conjugative transfer frequency in *Escherichia coli* (*E. coli*) through acting on the SdiA protein in quorum sensing system of *E. coli* [8]. Furthermore, Lu et al. found that silver nanoparticle (AgNP) and triclosan at environmentally relevant concentrations could facilitate the conjugative transfer of plasmid-borne ARGs across bacterial genera via regulating the oxidative stress and cell membrane permeability in bacteria [10,11]. Currently, considerable interest has centered on hormesis-based bacterial resistance of single agent, and the potential drug resistance (whether the facilitation of mutation or horizontal gene transfer) has been a significant indicator in the environmental risk evaluation of antibacterial agent [12,13]. However, there is little information regarding how multiple antibacterial agents interplay to induce the emergence and dissemination of ARGs. Antibacterial agents usually co-exist in environment [14], thus the pollution of bacterial resistance probably relates to the joint selection pressure of multiple antibacterial agents on the bacterial acquisition of ARGs. Furthermore, the co-existence of antibacterial agents may change the resistance risk of single agent. Therefore, it is of importance to judge the joint action of antibacterial agents on bacterial resistance, which will benefit the environmental risk assessment of mixed antibacterial agents and the investigation of bacterial resistance pollution.

Here, bacterial mutation and conjugative transfer (as the main way of horizontal gene transfer) are, respectively used to characterize the emergence and dissemination of ARGs [15], [16], [17]. Through obtaining key parameters in the dose-response of antibacterial agents on the mutation frequency and conjugative transfer frequency, the method for judging joint action of antibacterial agents on bacterial resistance was established according to the judgment of joint toxic action. This method included five basic procedures, and the specific steps in each procedure were described and explained in detail. Furthermore, each procedure was validated using the judgement of the joint resistance action for AgNP and quorum sensing inhibitor (QSI) as a case study.

### Method details: the five basic procedures (Fig. 1)

#### Procedure 1: toxicity determination

The single toxicity of each test antibacterial agent was investigated to obtain the toxicity parameters, which were used to design the mixture of antibacterial agents. Setting *E. coli* MG1655 K-12 (purchased from Biovector Co., Ltd. (Bejing, China)) as the model bacteria, the dose-response relationship of single antibacterial agent to the growth of *E. coli* was determined using microplate toxicity analysis.

**Step 1:** A preculture of *E. coli* was grown at 37 °C with 180 rpm shaking for 6 h (logarithmic growth period) in LB broth media (containing 1% (*w*/*v*) tryptone, 0.5% (*w*/*v*) yeast extract, and 1% (*w*/*v*) NaCl with the pH adjusted to 7.0–7.2), which was then diluted about 10^5^ times and used as an inoculum (approximately 5 × 10^4^ cell mL^−1^) in the following toxicity test. Tryptone was obtained from Sinopharm Chemical Reagent Company (Shanghai, China). Yeast extract (super pure) and NaCl (AR, purity 99.5%) were bought from Aladdin Industrial Corporation (Shanghai, China).
**Step 2:** Serial dilutions of the test antibacterial agent were set through fixing equal log dose interval between adjacent concentrations (usually 12–16 concentration points).

*Note: The lowest and largest concentration of test agent should be set based on the result in the preliminary experiment, which guarantee that the dose-response relationship covers the no effect (0) and the maximum inhibition.*

**Step 3:** 80 μL of test antibacterial agent, 80 μL of MH broth media (composed of 3% (*w*/*v*) beef infusion, 1.75% (*w*/*v*) casein hydrolysate, and 0.15% (*w*/*v*) water-soluble starch with the pH adjusted to 7.2–7.4), and 40 μL of the *E. coli* inoculum were added into each well of a transparent 100-well (or 96-well) microplate in order (total 200 μL for the treatment group). Meanwhile, the control group was obtained through substituting the 80 μL of test agent in the above 200 μL mixed solution with 80 μL of 1% (*w*/*v*) NaCl solution. All treatment and control groups had three or five parallels that independently carried out. Beef infusion, casein hydrolysate (approx. 70% free amino acids), and water-soluble starch (AR) were all purchased from Solarbio Life Sciences co., Ltd. (Beijing, China).
**Step 4:** The *OD*_600_ (optical density at 600 nm) of each well was determined on a microplate reader (Bioscreen C MBR, Growth Curves USA, Piscataway, NJ, USA) after the microplate was incubated at 37 °C with 180 rpm shaking for 24 h.
**Step 5:** The toxicity of test antibacterial agent to *E. coli* growth was expressed as the *Inhibition* (%) of *OD*_600_ value (Eq. (1)):
  (1) $$Inhibition\;(\%)\;=\;\frac{OD_{600,c\;}-\;OD_{600,t}}{OD_{600,c}}\;\times \;100\%$$

where *OD*_600,_*_c_* and *OD*_600,_*_t_* were the average *OD*_600_ values of the control and treatment groups, respectively. Then, the dose-response relationship of agent concentration to the corresponding inhibition of *E. coli* growth was obtained, and the standard deviation was used to represent for the error bar.

#### Procedure 2: mutation frequency determination

**Step 1:** The above microplate toxicity analysis in toxicity determination step was repeated in mutation test.
**Step 2:** The 200 μL solution in three or five paralleled wells for each treatment or control group were collected into a 1.5 mL centrifuge tube. *E. coli* in this collected solution was washed thrice with 1% (*w*/*v*) NaCl solution and then suspended in the same saline solution.

*Note: The process of washing E. coli included centrifuging for 3* min *with 6000* *rpm, removing the supernatant, shaking for 5 s using a vortex mixer, and adding 1* *mL 1% (w/v) NaCl solution.*

**Step 3:** Some of the *E. coli* suspension (usually at most 10 μL) was diluted about 10^6^ times with 1% (*w*/*v*) NaCl solution, which was used to determine the number of total *E. coli* in the suspension. Some of the *E. coli* suspension (usually the rest solution) was concentrated about 3 times, which was used to determine the number of mutant *E. coli* in the suspension.
**Step 4:** The diluted suspension and concentrated suspension were, respectively incubated on antibiotic-free and rifampicin-added (25 mg L^−1^) solid plate media (LB broth with 1.5–2% agar) for 12–15 h, and the number of total *E. coli* and mutant *E. coli* were obtained by counting the colony-forming units per milliliter of culture (CFU mL^−1^) in these two kinds of solid plates. Rifampicin (purity 97%) was obtained from Aladdin Industrial Corporation (Shanghai, China). Agar was bought from Sinopharm Chemical Reagent Company (Shanghai, China).

*Note: In Step 3 and 4, the diluted and concentrated ratio of suspension, the concentration of rifampicin, and the culture period of solid plate are not fixed, which could be adjusted according to the density of CFU in the preliminary experiment (convenient and clear to count).*

**Step 5:** The mutation frequency (*MF*) of *E. coli* in treatment and control groups were all obtained via calculating the ratio of CFU value for mutant *E. coli* to CFU value for total *E. coli* (Eq. (2)):
  (2) $$MF\;=\;\frac{\text{CF}U_{mE}}{\text{CF}U_{tE}}$$

where CFU*_mE_* and CFU_*tE*_, respectively referred to the average CFU values of mutant *E. coli* and total *E. coli* in each group. There were at least two duplicates for each group. The influence of test antibacterial agent on the mutation frequency of *E. coli* was expressed as the *Promotion* (%) of *MF* value (Eq. (3)):

(3) $$Promotion\;(\%)\;=\;\frac{MF_{t}\;-\;MF_{c}}{MF_{c}}\;\times \;100\%$$

where *MF_t_* and *MF_c_*, respectively indicated the average MF values of the treatment and control groups. Then, the dose-response relationship of agent concentration to the corresponding promotion of *MF* value was obtained, and the standard deviation was used to represent for the error bar.

#### Procedure 3: conjugative transfer frequency determination

RP4 plasmid conjugative transfer between *E. coli* (RP4) strain and *E. coli* (Nal) strain was used as the research model for investigating the conjugative transfer of ARGs. The *E. coli* (RP4) strain that harbored the RP4 plasmid (carrying the ampicillin, kanamycin, and tetracycline resistance genes) was used as the donor, and the *E. coli* (Nal) strain that was resistant to nalidixic acid was used as the recipient.

**Step 1:***E. coli* (RP4) and *E. coli* (Nal) were cultured at 37 °C with 180 rpm shaking for 6 h to logarithmic growth period in LB broth media that containing corresponding antibiotics.
**Step 2:** The cultured *E. coli* (RP4) and *E. coli* (Nal) were washed thrice with 1% (*w*/*v*) NaCl solution, the OD_600_ value of which were then both adjusted to 0.5 with the same saline solution.

*Note: The process of washing E. coli (RP4) and E. coli (Nal) included centrifuging for 3* min *with 6000* *rpm, removing the supernatant, shaking for 5 s using a vortex mixer, and adding 1* *mL 1% (w/v) NaCl solution.*

**Step 3:***E. coli* (RP4) and *E. coli* (Nal) were both shaken for 20 min with 180 rpm, and then mixed at a ratio of 1:2 to obtain a mixed inoculum. After 180 rpm shaking for 20 min, the final inoculum (approximately 5 × 10^6^ cell mL^−1^) was obtained. Serial dilutions of the test antibacterial agent were set through fixing equal log dose interval between adjacent concentrations (usually 10–12 concentration points).
*Note: The lowest and largest concentration of test agent should be set based on the result in the preliminary experiment, which guarantee that the dose-response relationship exhibits inverted U shape and the lowest concentration of test agent has no effect (0).*
**Step 4:** 40 μL of test antibacterial agent, 100 μL of 2-fold LB broth media, and 60 μL of the inoculum for mixed bacteria were added into a 1.5 mL centrifuge tube in order (total 200 μL for the treatment group). Meanwhile, the control group was obtained through substituting the 40 μL of test agent in the above 200 μL mixed solution with 40 μL of 1% (*w*/*v*) NaCl solution.
**Step 5:** After a 12 h mating period at 37 °C, the suspension in the centrifuge tube was diluted about 10^5^ times with 1% (*w*/*v*) NaCl solution. The diluted suspension was then incubated on both solid plate media (LB broth with 1.5–2% agar) containing 40 mg mL^−1^ of nalidixic acid and solid plate media (LB broth with 1.5–2% agar) containing 40 mg mL^−1^ of nalidixic acid and 40 mg mL^−1^ of kanamycin, and the number of recipients and transconjugants were measured by counting CFU mL^−1^ in these two kinds of solid plates. Nalidixic acid (purity 98%) and kanamycin (purity 98%) were both purchased from Aladdin Industrial Corporation (Shanghai, China).
*Note: The diluted ratio of suspension and the concentration of nalidixic acid and kanamycin are not fixed, which could be adjusted according to the density of CFU in the preliminary experiment (convenient and clear to count).*
**Step 6:** The conjugative transfer frequency (*CTF*) of RP4 plasmid in treatment and control groups were all obtained via calculating the ratio of CFU value for transconjugants to CFU value for recipients (Eq. (4)):
  (4) $$CTF\;=\;\frac{\text{CF}U_{t}}{\text{CF}U_{r}}$$

where CFU*_t_* and CFU*_r_*, respectively represented the average CFU values of transconjugants and recipients. There were at least two duplicates for each group. The influence of test antibacterial agent on the conjugative transfer frequency of RP4 plasmid was expressed as the *Promotion* (%) of *CTF* value (Eq. (5)):

(5) $$Promotion\;(\%)\;=\;\frac{CTF_{t}\;-\;CTF_{c}}{CTF_{c}}\;\times \;100\%$$

where *CTF_t_* and *CTF_c_*, respectively referred to the average *CTF* values of the treatment and control groups. Then, the dose-response relationship of agent concentration to the corresponding promotion of *CTF* value was obtained, and the standard deviation was used to represent for the error bar.

#### Procedure 4: data fitting, key parameters obtaining, and antibacterial mixture designing

**Step 1:** The toxic dose-response relationship of single antibacterial agent to the growth of *E. coli* was fitted via two kinds of regression function: (a) the built-in DoseResp regression function in Origin software (version 86, OriginLab Corporation, Northampton, MA, USA) was used when there was no hormetic effect in the dose-response relationship; (b) the artificially constructed Hormesis regression function in Origin software was used when there was a hormetic effect in the dose-response relationship. The fitting equation for Hormesis regression function (Eq. (6)) was as follows [18]:
  (6) $$y\;=\;C\;-\;\frac{D\;+\;m}{1\;+\;{\left(\frac{x}{a}\right)}^{b}}\;+\;\frac{D\;-\;C\;+\;m}{1\;+\;{\left(\frac{x}{p}\right)}^{q}}$$

where *x* was the concentration of test antibacterial agent, and *y* was the corresponding inhibitory effect; *C* and *D* represented the inhibitory rates corresponding to the top asymptote and the bottom asymptote, respectively; *m* referred to the stimulatory rate corresponding to the stimulation asymptote; *a* and *b* indicated the first median and slope in the stimulatory region, respectively; *p* and *q* were the median and slope in the inhibitory region, respectively. In the mutation and conjugative transfer test, the observed dose-response data of single antibacterial agents were fitted through the inverted Hormesis regression function.

Note: The Hormesis regression function and its inverted one should be input into Origin software by users, and complied via *the program with the corresponding parameters precisely defined. When conducting the fitting process, a certain value should be artificially given to each parameter in the Hormesis regression function based on the actual dose-response relationship of single agent.*

**Step 2:** According to the fitted toxic dose-response curve of single antibacterial agent to the growth of *E. coli*, the median effective concentration of each agent (EC_50_) was obtained to characterize its toxicity. The mixture of antibacterial agents used in mutation and conjugative transfer test was designed based on the equitoxic ratio of EC_50_ for each single agent [19]. Serial dilutions of the mixed antibacterial agents were set through fixing equal log dose interval between adjacent concentrations.

Note: The design of antibacterial mixture is not fixed, and the concentration of each agent and their mixing *ratio could be adjusted based on actual needs. The lowest and largest concentration of mixed agents should be set based on the result in the preliminary experiment, which guarantee that the dose-response relationship exhibits inverted U shape and the lowest concentration of mixed agents has no effect (0).*

**Step 3:** Then, the impacts of mixed antibacterial agents on mutation frequency and conjugative transfer frequency of RP4 plasmid in *E. coli* were determined using the same method as mentioned in Procedure 1–3. The corresponding dose-response data were fitted through the inverted Hormesis regression function.

Note: When conducting the fitting process, a certain value should be artificially given to each parameter in *the inverted Hormesis regression function based on the actual dose-response relationship of mixed agents.*

**Step 4:** To characterize the facilitation of single and mixed antibacterial agents on the mutation and conjugative transfer frequency, some key parameters should be selected in the corresponding fitted dose-response curves. Here, the initial (minimum) concentrations that single and mixed agents began to promote the mutation and conjugative transfer frequency were set as the key parameters, which could be regarded as the risk thresholds in inducing the emergence or dissemination of ARGs. Compared with the concentration that provoked initial 0% or 5% promotion, the concentration that provoked initial 10% promotion in the actual regression curve was more appropriate to be the risk threshold because it was easier to observe and accurately determine. Thus, the concentrations that induced initial 10% promotion on the mutation and conjugative transfer frequency in the regression curves, i.e., MC_10_ and CTC_10_, were obtained to feature the facilitation of single and mixed antibacterial agents on bacterial resistance.

#### Procedure 5: judgement of joint resistance action

**Step 1:** Toxic unit (*TU*) method has been widely used to judge the joint toxic action among the components in the mixed antibacterial agents, in which the *TU* value is calculated via the EC_50_ of the components and the concentrations of components when the mixture is at EC_50_ [20]. According to *TU* method, the joint resistance action of test antibacterial agents was judged via calculating the mutation unit (*MU*) and the conjugative transfer unit (*CTU*), and the corresponding Eqs. (7) and (8) were as follows:
  (7) $$MU\;=\sum_{i=1}^{n}\frac{c_{i}}{MC_{10i}}\;$$
  (8) $$CTU=\sum_{i=1}^{n}\frac{c_{i}^{\prime }}{\text{CT}C_{10i}}$$

where *c_i_* and *c_i_′* represented the concentrations of component *i* when the mixture of antibacterial agents were at the concentrations of MC_10_ and CTC_10_ (MC_10mix_ and CTC_10mix_), respectively; MC_10_*_i_* and CTC_10_*_i_* referred to the MC_10_ and CTC_10_ values of component *i* when acting alone, respectively. Based on the 10% promotion effect and the fixed ratio of agents in the mixture, these values were obtained from the dose-response curves of single and mixed agents to mutation and conjugative transfer frequency.

**Step 2:** In *TU* method, the joint toxic action of antibacterial agents could be judged to be antagonism, addition, or synergism via comparing *TU* value with 0.8 and 1.2 [21,22]. Similarly, the joint resistance action of antibacterial agents could be judged as follows: when *MU* (*CTU*) < 0.8, the joint action of components on the promotion of mutation frequency (conjugative transfer frequency) was synergism; when 0.8 ≤ *MU* (*CTU*) ≤ 1.2, the joint action of components on the promotion of mutation frequency (conjugative transfer frequency) was addition; and when *MU* (*CTU*) > 1.2, the joint action of components on the promotion of mutation frequency (conjugative transfer frequency) was antagonism.

## Method validation

As the possible antibiotic alternatives, AgNP and QSI have the great potential in preventing and treating microbial infections [23,24]. Over the past two decades, AgNP and QSI have been used in medicine, hygiene, cosmetics, and breeding industry [25,26]. Due to the possible coexistence of these two antibacterial agents in the environment, it is necessary to explore how AgNP and QSI interplay on the bacterial resistance, i.e., to judge their joint resistance action, which will benefit evaluating the resistance risk for the combined exposure of AgNP and QSI.

The joint resistance action of AgNP and QSI was judged using the above method (the data originated from Shen et al. 2021), and the five basic procedures were validated (Fig. 2) [15]. The test AgNP (5 nm) was purchased from Huzheng Nano Technology Co., Ltd (Shanghai, China). 1-pyrrolidino-1-cyclohexene (1P1C), as a typical QSI, which was all obtained from Sigma-Aldrich Co., Ltd. (St. Louis, MO, USA) which without repurification (purity ≥ 99%). First, the influence of single test AgNP and 1P1C on the growth, mutation frequency, and RP4 plasmid conjugative transfer frequency of *E. coli* were investigated according to **Procedure 1–3**, and the results were depicted in Fig. 2**a**. Second, because there was no hormetic phenomenon in the dose-response relationship of each test agent to *E. coli* growth, the DoseResp regression function was applied to fit these dose-response data (**Procedure 4**), as shown in Fig. 2**b**. It was obtained from these fitted curves that the EC_50_ values of AgNP and 1P1C were 1.50E-06 and 7.42E-03 mol L^−1^, respectively. Third, the binary mixture of AgNP and 1P1C was prepared, in which the ratio of component AgNP to 1P1C was equal to EC_50(AgNP):_ EC_50(QSI)_ (**Procedure 4**), and the impact of AgNP-1P1C mixture on the mutation frequency and RP4 plasmid conjugative transfer frequency was determined, and the results were displayed in Fig. 2**c**. Fourth, the observed dose-response data of AgNP, 1P1C, and their binary mixture on the mutation frequency and RP4 plasmid conjugative transfer frequency were fitted through the inverted Hormesis regression function (depicted in Fig. 2**d**). As described in **Procedure 4**, MC_10_ and CTC_10_ values for AgNP, 1P1C, and their binary mixture were obtained from the corresponding regression curves. Finally, the *MU* and *CTU* values for the binary mixture of AgNP and 1P1C were calculated on the basis of the equations in **Procedure 5**, as shown in Fig. 2**e**. It was obvious that the *MU* value (64.27) and *CTU* value (5.10) were all larger than 1.2, indicating that the joint resistance action between AgNP and 1P1C exhibited antagonistic effect. The results implied that although AgNP and QSI could facilitate the emergence and dissemination of ARGs, the coexistence of AgNP and QSI may largely mitigate the resistance risk induced by the single agent.

**Fig. 1:**
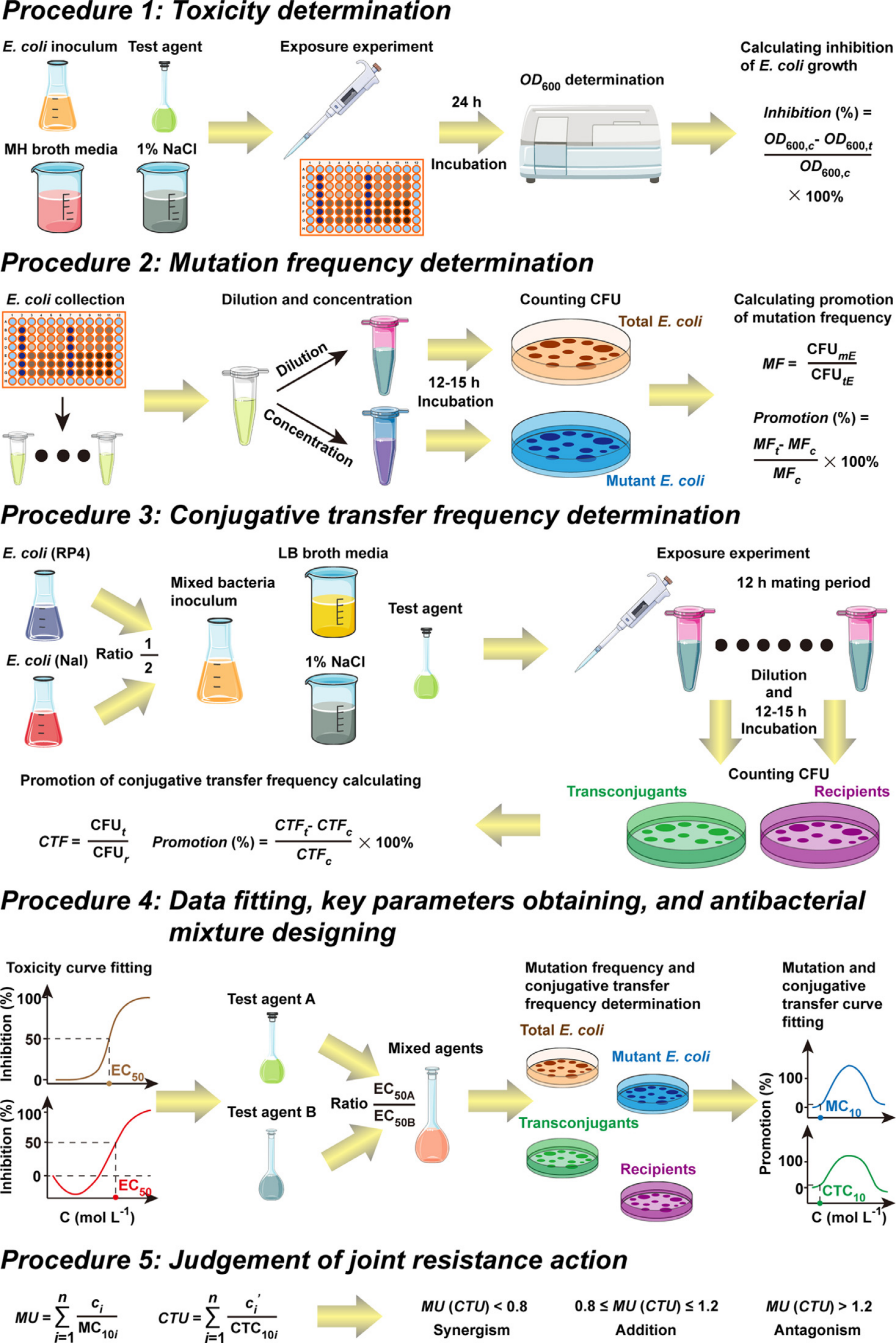
Schematic representation of the five basic procedures in the method for judging joint resistance action of antibacterial agents.

**Fig. 2:**
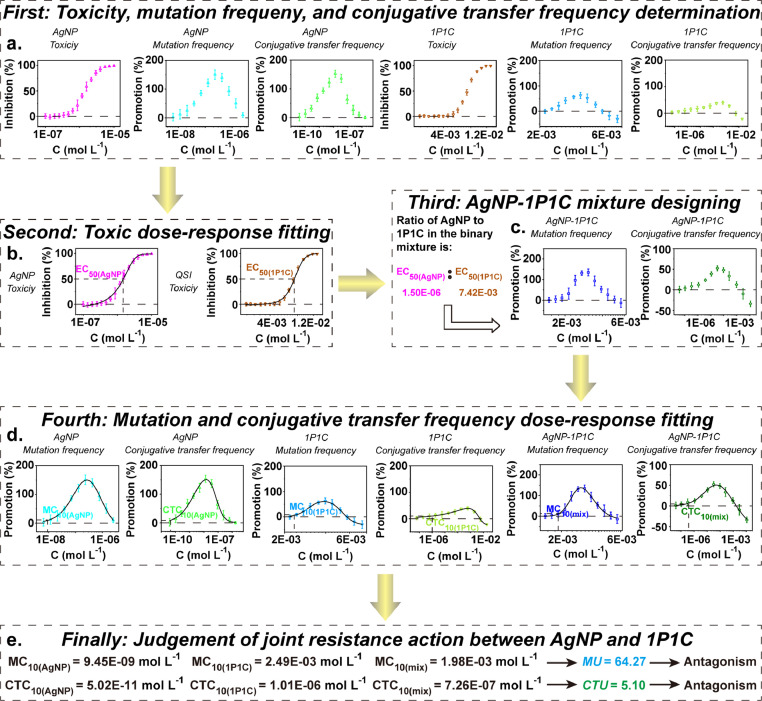
The judgment of joint resistance action of AgNP and QSI using the developed method. Source: Agathokleous et al. 2021 [13].

## Conclusion

This study introduced five procedures for evaluating the joint resistance action of binary or multicomponent antibacterial agents under the laboratorial level. This method can help to fast assess the potential resistance risk for the mixture of antibacterial agents through experimental operation and mathematical calculation, which can be used in environmentology, toxicology, pharmacology, biology, and medicine. In addition, this method can also guide the mechanistic explanation for the facilitation of mixed antibacterial agents on bacterial resistance, the environmental risk assessment and clinical use of combined antibacterial agents.
